# Supplementary material for: Cryo-EM structure of the human COP1-DET1 ubiquitin ligase complex
Source: Nat Commun. 2026 Jan 15;17:543. doi: 10.1038/s41467-026-68375-7 (PMC12808801; doi:10.1038/s41467-026-68375-7)
Supplement: Supplementary file 1 — Supplementary Information [file 41467_2026_68375_MOESM1_ESM.pdf]

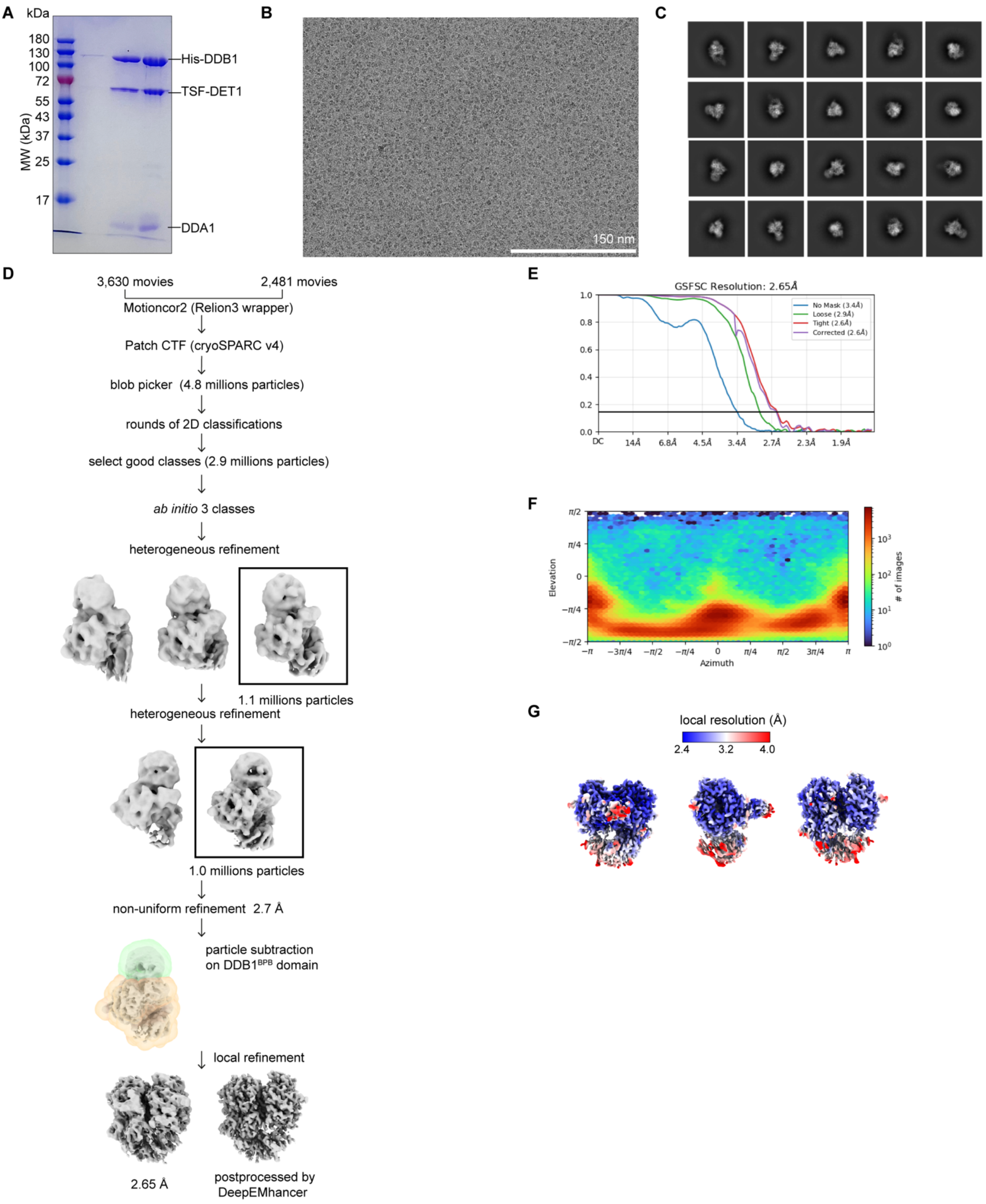

Supplementary Figure 1. Cryo-EM analysis of the DDB1-DDA1-DET1 (DDD) complex.

A, Coomassie blue-stained SDS-PAGE analysis of the purified DDB1-DDA1-DET1 (DDD) complex. MW, molecular weight. TSF, twin-strep-FLAG. Source data are provided as a Source Data file. **B**, Representative cryo-EM micrograph of the DDD complex. Scale bar, 150 nm. **C**, Representative 2D class

averages of the DDD complex. **D**, Flow chart showing cryo-EM data processing steps. **E**, Fourier shell correlation (FSC) plots between two independently refined half-maps, calculated with no mask (blue), loose mask (green), tight mask (red), and mask-corrected (purple). A cut-off of 0.143 (black line) was used to estimate the resolution. **F**, Angular distribution of particle projections calculated in cryoSPARC. The heatmap shows the number of particles for each viewing angle. **G**, Local resolution map of the DeepEMhancer post-processed cryo-EM reconstruction, colored according to the indicated scale.

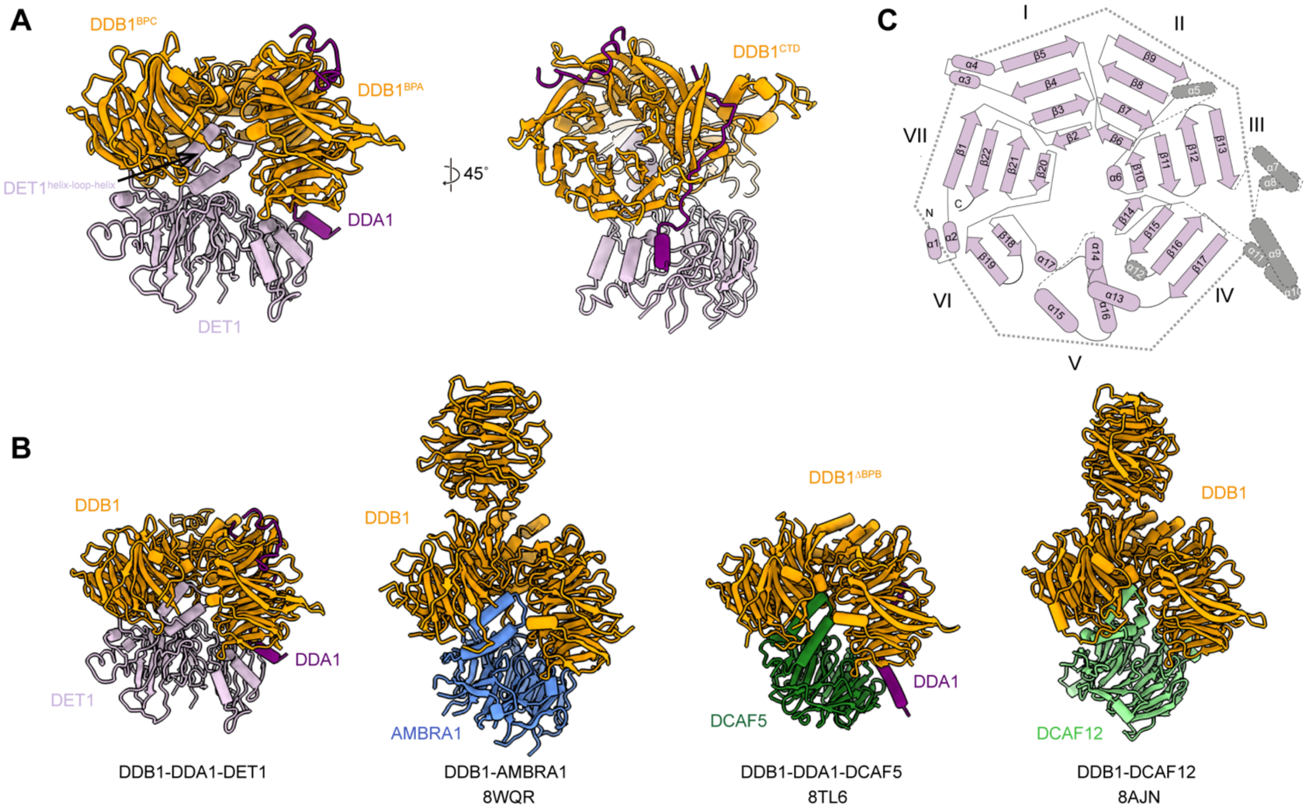

Supplementary Figure 2. Comparison of DET1 and other DCAFs bound to DDB1.

A, Molecular model of the DDB1$^{\Delta BPB}$-DET1-DDA1 complex shown in two views. Subunits are colored as follows: DDB1 (orange), DDA1 (purple), and DET1 (thistle). **B**, Multiple DCAFs, including DET1, AMBRA1 (PDB: 8WQR), DCAF5 (PDB: 8TL6) and DCAF12 (PDB: 8AJN) bind to the same pocket on DDB1. **C**, Cartoon representation of the DET1 structure.

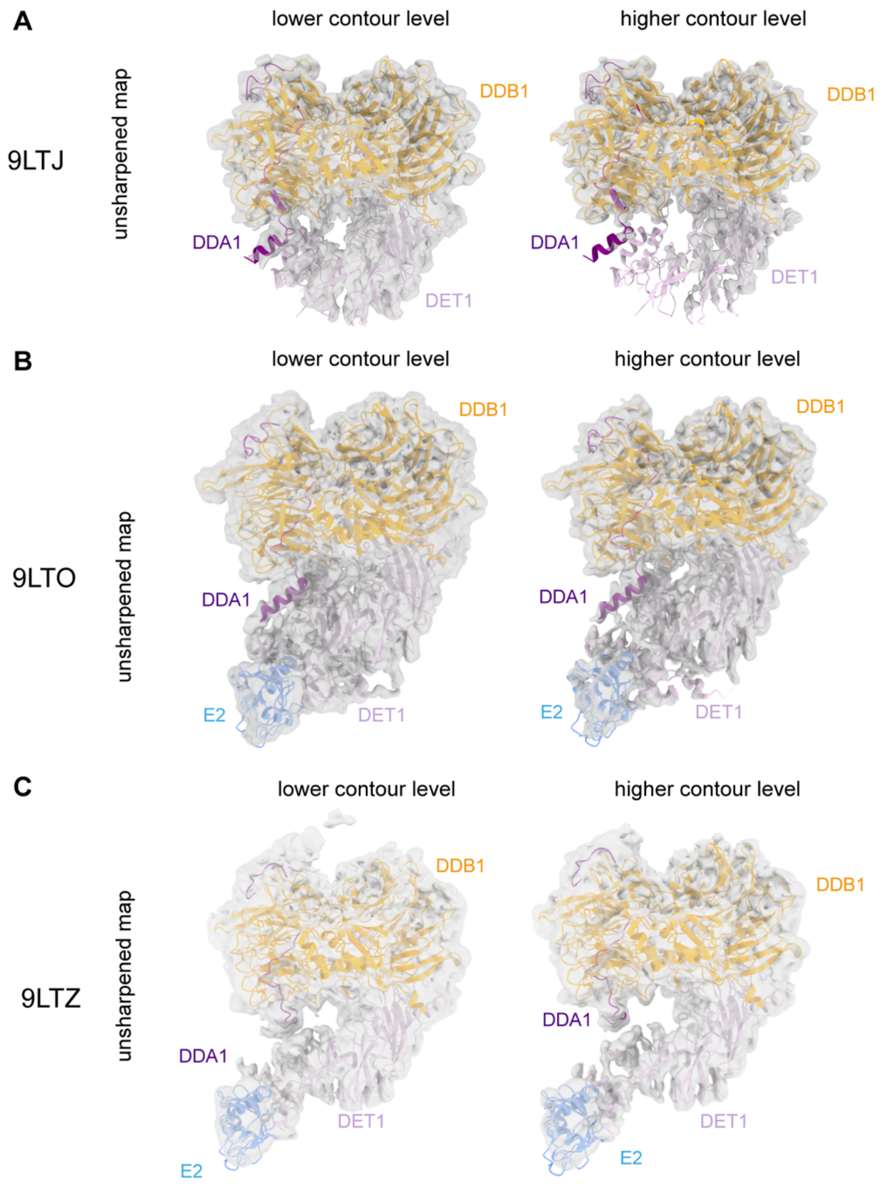

Supplementary Figure 3. Comparison of the unsharpened map of DDD and DDD-E2 complexes. **A-C**, Comparison of the unsharpened maps of DDD (9LTJ, EMD-63371) and DDD-E2 (9LTO, EMD-63374; 9LTZ, EMD-63385). Maps are shown at both lower and higher contour levels to account for the flexibility of DET1 subunit.

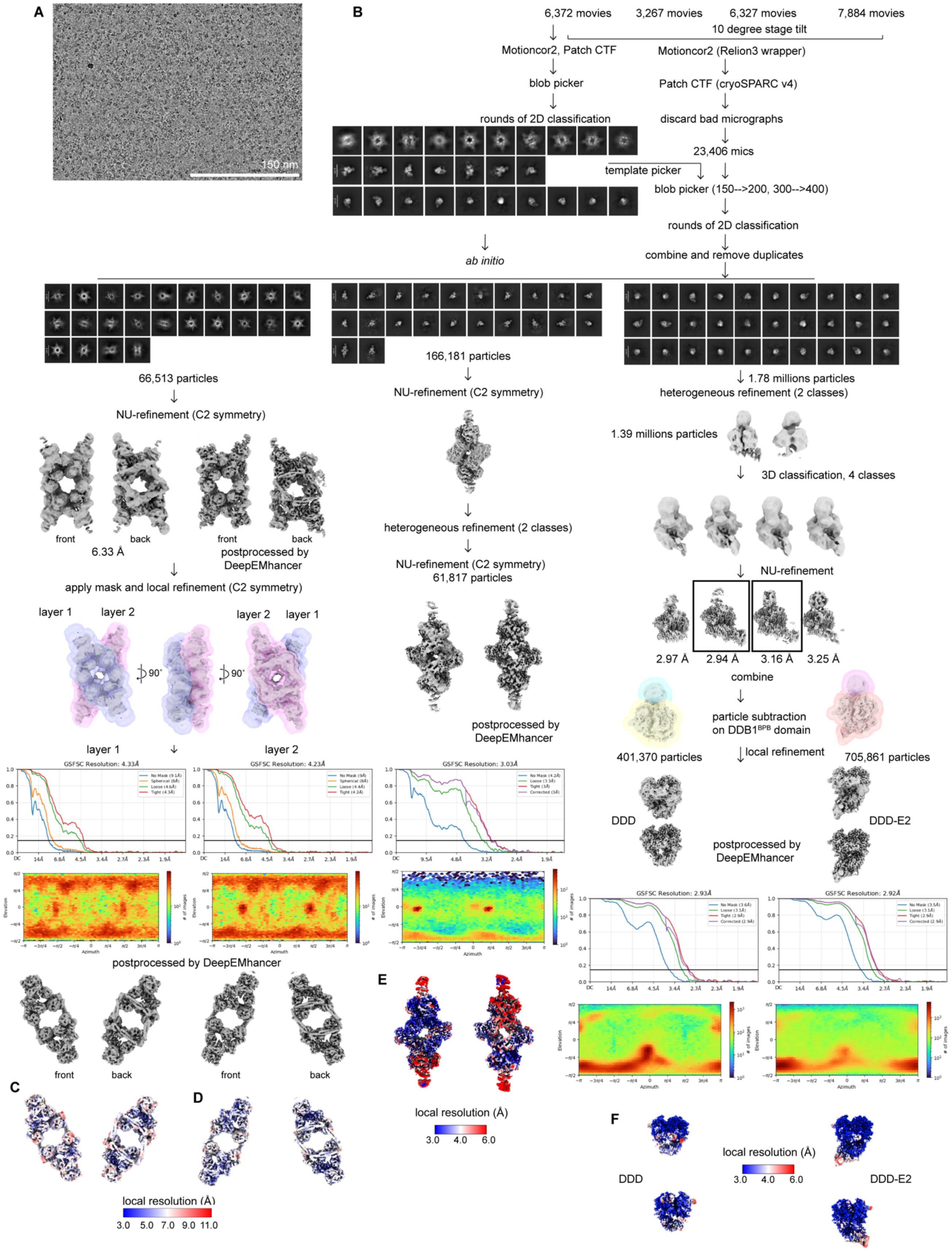

Supplementary Figure 4. Cryo-EM structure determination of the DDB1-DDA1-DET1-COP1 complex.

A, Representative cryo-EM micrograph of the DDB1-DDA1-DET1-COP1 complex. Scale bar, 150 nm. **B**, Flow chart of cryo-EM data processing. NU-refinement: non-uniform refinement. **C-D**, DeepEMhancer post-processed cryo-EM maps of the stacked DDD-COP1 complex, color-coded according to local resolution estimation. **E**, DeepEMhancer post-processed cryo-EM map of the dimeric DDD-COP1-E2 (Ube2e2) complex, color-coded by local resolution. **F**, DeepEMhancer post-processed cryo-EM maps of the DDD and DDD-E2 complexes, with local resolution coloring.

A

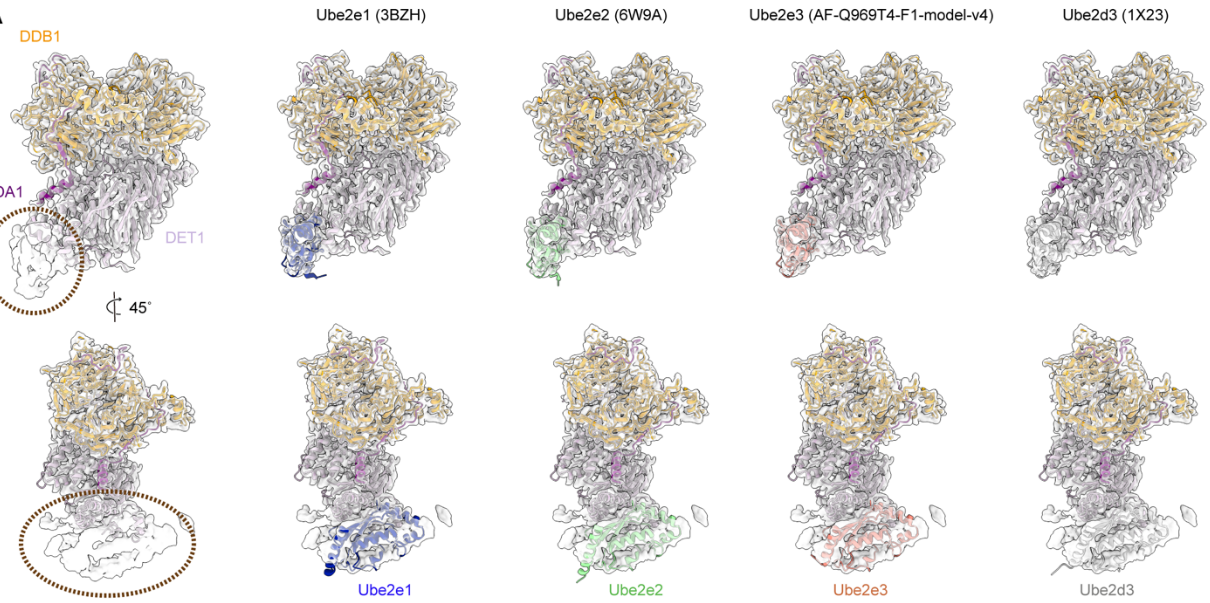

B

Description	Sum PEP Score	Coverage [%]	# Peptides	# PSMs	# Unique Peptides	# AAs	MW [kDa]	Score Sequest HT: Sequest HT	# Peptides (by Search Engine): Sequest HT
DNA damage-binding protein 1	1994.441	95	572	813	572	1140	126.9	2153.6	572
E3 ubiquitin-protein ligase COP1	694.394	89	211	279	211	731	80.4	732.53	211
DET1	321.65	71	106	137	106	550	63.8	284.16	106
DET1- and DDB1-associated protein 1 (DDA1)	108.062	97	34	54	34	102	11.8	104.32	34
Ubiquitin-conjugating enzyme E2 E1 (UBE2E1)	82.47	76	32	43	6	193	21.4	78.7	32
Ubiquitin-conjugating enzyme E2 E2 (UBE2E2)	70.885	72	28	37	2	201	22.2	64.71	28
Ubiquitin-conjugating enzyme E2 E3 (UBE2E3)	62.626	63	24	30	1	207	22.9	61.44	24
Ubiquitin-conjugating enzyme E2 D3 (UBE2D3)	45.505	76	12	19	12	147	16.7	31.09	12

C

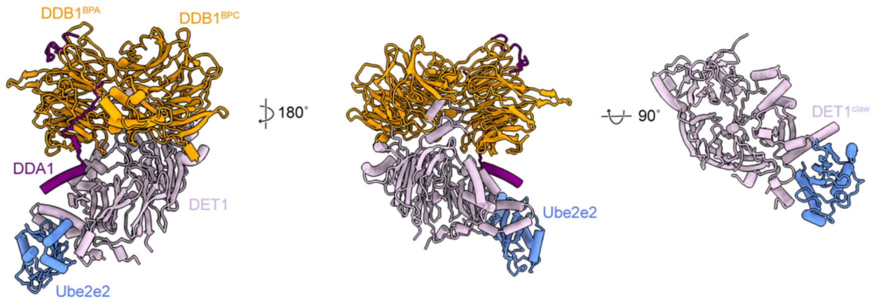

D

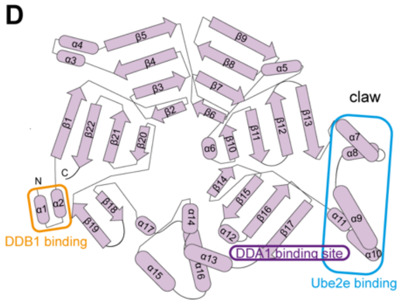

Supplementary Figure 5. DET1 binds the Ube2e family.

A, Fitting of the core UBC domains from the Ube2e family and Ube2d3 into the DDD-E2 complex density. Structures used include Ube2e1 (PDB: 3BZH), Ube2e2 (PDB: 6W9A), Ube2e3 (AlphaFold2 predicted model), and Ube2d3 (PDB: 1X23). The presented map was post-processed with DeepEMhancer. **B**, Proteins associated with the DDB1-DDA1-DET1-COP1 complex, purified from Expi293F cells, were identified by mass spectroscopy. **C**, Molecular model of the DDD-Ube2e2 complex shown from multiple viewpoints. **D**, Cartoon representation of DET1 from the DDD-E2 structure, highlighting its interaction regions with DDA1, DDB1 and Ube2e2.

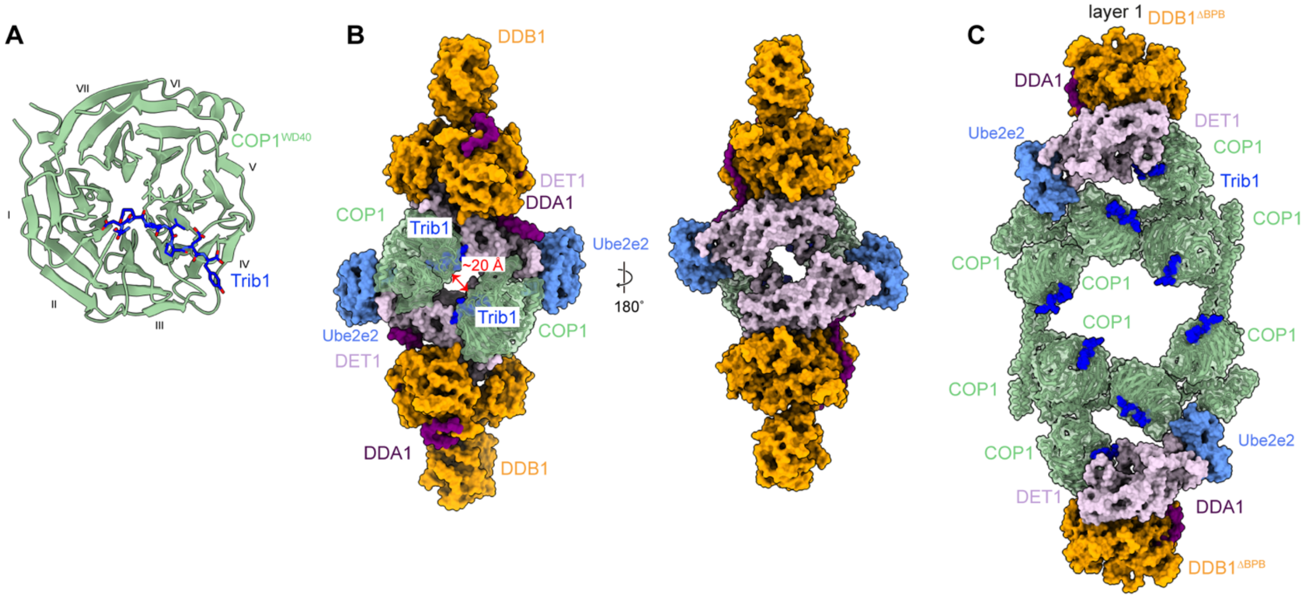

Supplementary Figure 6. Mapping substrate binding sites in the dimeric and stacked assemblies of DDB1-DDA1-DET1-Ube2e2-COP1 complexes.

A, The model of COP1^{WD40}-Trib1 (PDB: 5IGQ), showing the binding between the VP motif of Trib1 and the binding pocket of COP1. **B-C**, Docking of Trib1 VP peptides onto the dimeric DDD-Ube2e2-COP1 structure (**B**) and the layer 1 of the stacked DDD-Ube2e2-COP1 structure (**C**) reveal the substrate-binding sites. Trib1 VP motifs are shown as blue spheres.

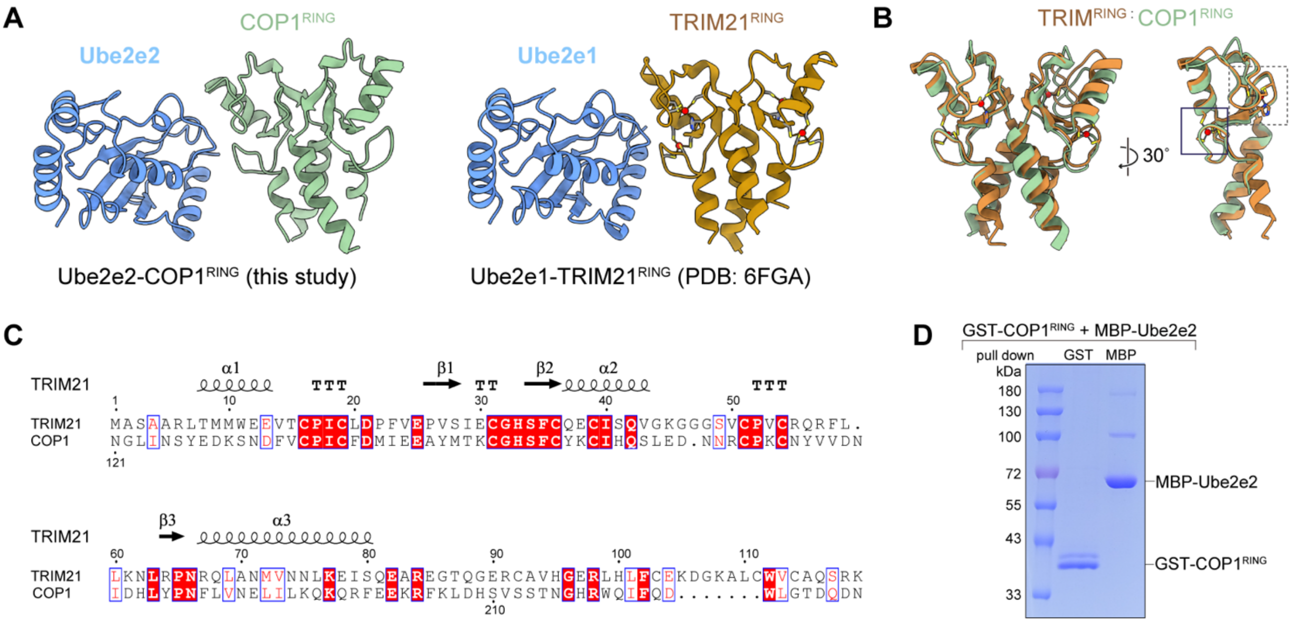

Supplementary Figure 7. Comparison between COP1[RING] and TRIM21[RING].

A, Structure comparison between the Ube2e2-COP1[RING] (*left*) and the Ube2e1-TRIM21[RING] (*right*) complexes. **B-C**, Structural and sequence alignment between the COP1[RING] and TRIM21[RING] domains. **D**, Pull-down experiment of GST-COP1[RING] with MBP-Ube2e2. Cell lysates were divided in two fractions and incubated separately with GST and MBP beads. The eluents were analysed by SDS-PAGE. Data in D are representative of three independent experiments. Source data are provided as a Source Data file.

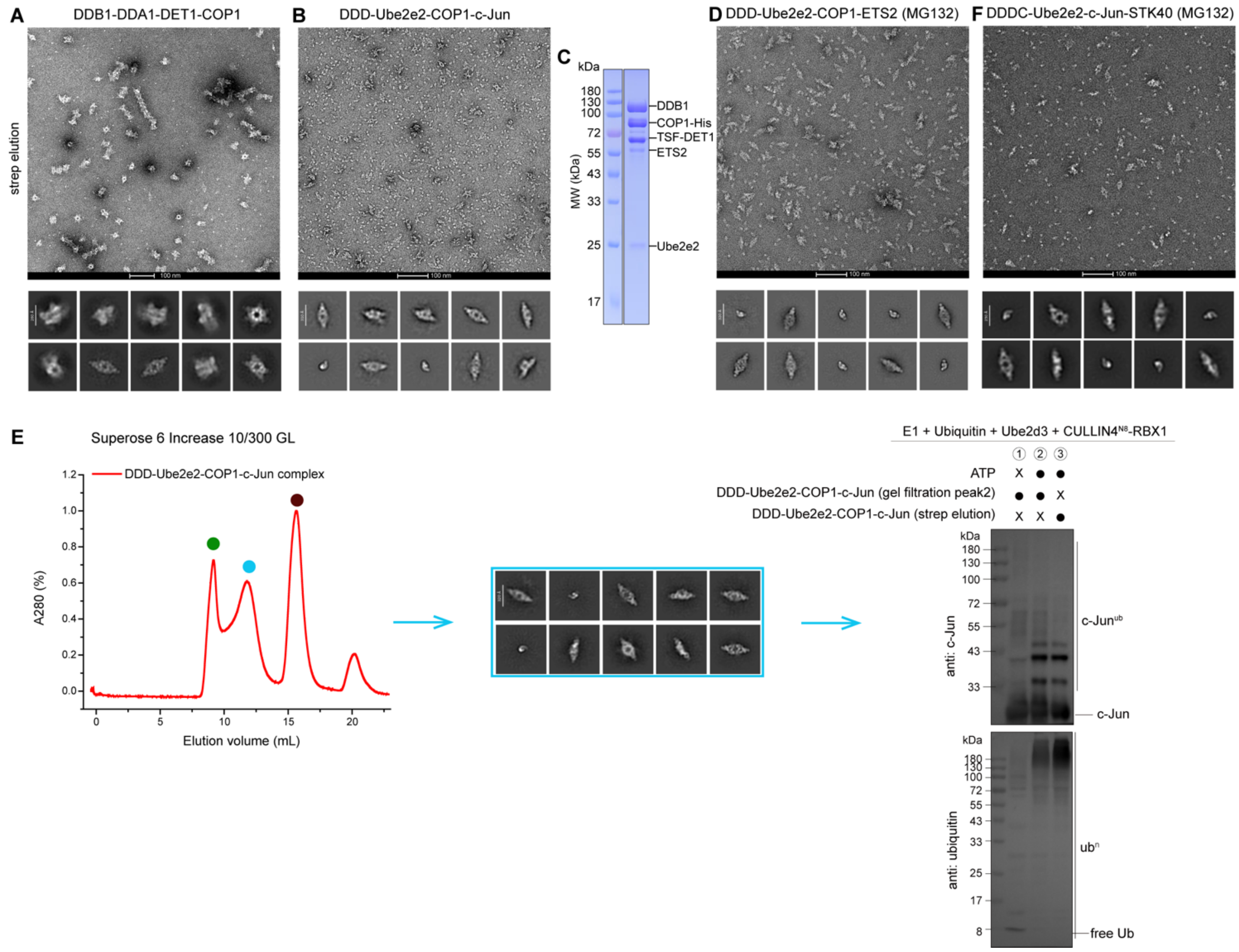

Supplementary Figure 8. Analysis of the DDD-COP1 complex and substrate/adaptor co-expression effects assessed by negative staining. Scale bar, 100 nm.

A-B, The negative stain micrograph and 2D class averages of the DDD-COP1 complex (**A**) and the DDD-Ube2e2-COP1-c-Jun complex (**B**). **C**, Coomassie blue-stained SDS-PAGE analysis of the purified DDD-Ube2e2-COP1-ETS2 complex. Source data are provided as a Source Data file. **D**, The negative stain image and 2D class averages of the DDD-Ube2e2-COP1-ETS2. **E**, Strep-eluted DDD-Ube2e2-COP1-c-Jun complex, or dimeric complex purified by Superose 6 size exclusion chromatography were incubated with E1 (Uba1), ubiquitin, Ube2d3 and neddylated CULLIN4-RBX1. Immunoblotting with antibodies against c-Jun and ubiquitin was performed, with the presence (●) or absence (x) of each component indicated. The experiments were repeated independently three times with similar results. **F**, The negative stain image and 2D class averages of the DDD-Ube2e2-COP1-c-Jun-STK40 complex. MG132 was included during transfection for both complexes to inhibit proteasomal degradation. Source data are provided as a Source Data file.

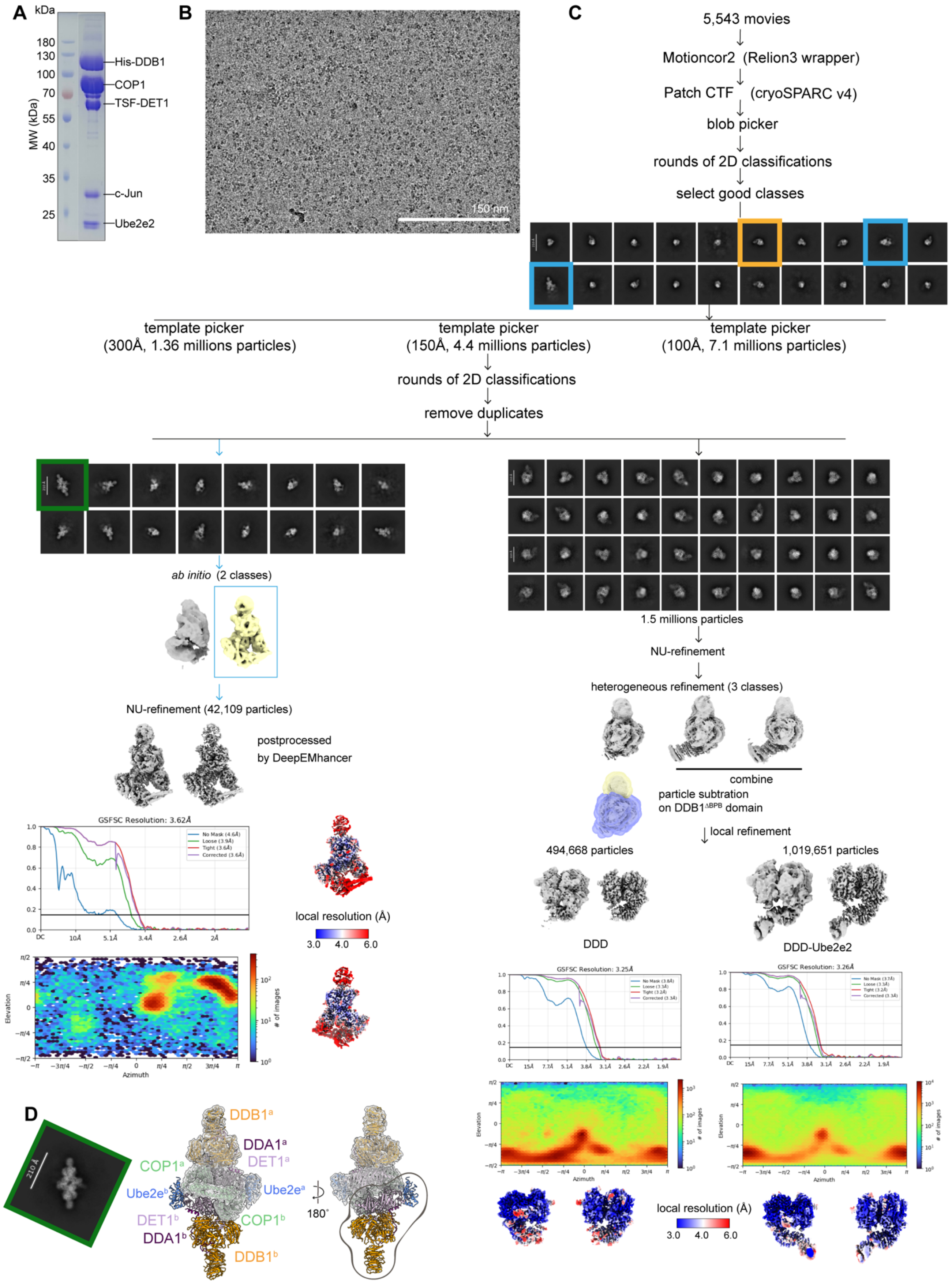

A
kDa
180
130
100
70
55
40
35
25
MW (kDa)
His-DDB1
COP1
TSF-DET1
c-Jun
Ube2e2
B
150 nm
C
5,543 movies
Motioncor2 (Relion3 wrapper)
Patch CTF (cryoSPARC v4)
blob picker
rounds of 2D classifications
select good classes
template picker (300Å, 1.36 millions particles)
template picker (150Å, 4.4 millions particles)
template picker (100Å, 7.1 millions particles)
rounds of 2D classifications
remove duplicates
ab initio (2 classes)
NU-refinement (42,109 particles)
postprocessed by DeepEMhancer
GSFSC Resolution: 3.62Å
local resolution (Å)
3.0 4.0 6.0
Elevation
Azimuth
# of images
1.5 millions particles
NU-refinement
heterogeneous refinement (3 classes)
combine
particle subtration on DDB1ΔBPB domain
local refinement
494,668 particles
1,019,651 particles
DDD
DDD-Ube2e2
GSFSC Resolution: 3.25Å
GSFSC Resolution: 3.26Å
local resolution (Å)
3.0 4.0 6.0
D
210 Å
DDB1a
DDA1a
DET1a
COP1a
Ube2eb
Ube2ea
DET1b
COP1b
DDA1b
DDB1b
180˚

Supplementary Figure 9. Cryo-EM structure determination of the DDB1-DDA1-DET1-Ube2e2-COP1-c-Jun complex.

A, Coomassie blue-stained SDS-PAGE analysis of the purified DDB1-DDA1-DET1-Ube2e2-COP1-c-Jun complex. MW, molecular weight. TSF, twin-strep-FLAG. Source data are provided as a Source Data file. **B**, Representative cryo-EM micrograph of the DDB1-DDA1-DET1-Ube2e2-COP1-c-Jun complex. Scale bar, 150 nm. **C**, Flow chart of cryo-EM data processing. NU-refinement, non-uniform refinement. **D**, The 2D class average demonstrating the dimeric assembly of the DDD-Ube2e2-COP1-c-Jun complex, with a chimera model generated by applying C2 symmetry to the experimental coordinate file. The region corresponding to DDD, which was not resolved and was built by symmetry, was indicated by a circle.

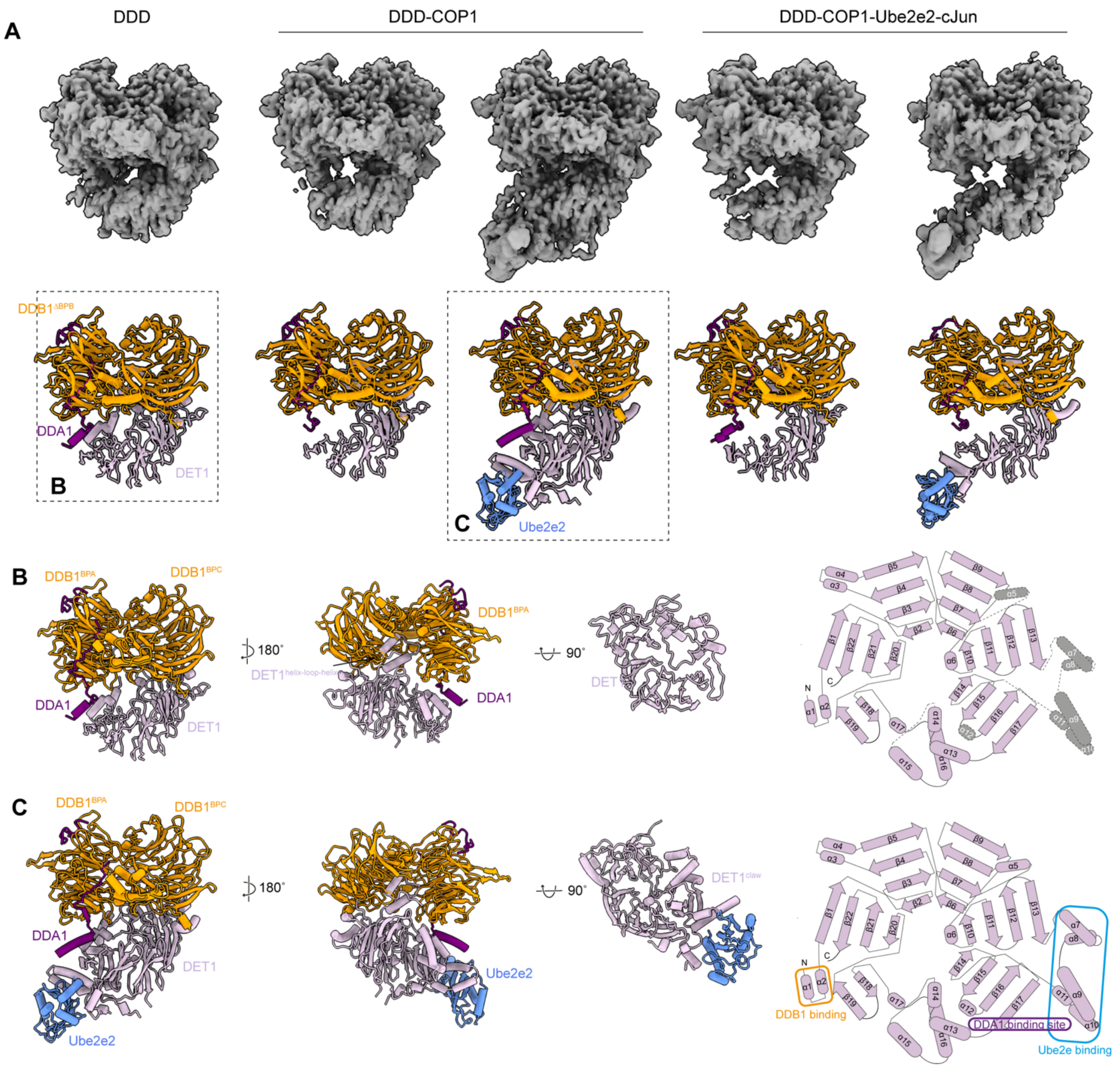

Supplementary Figure 10. Comparison of the DDD and the DDD-Ube2e2 complexes obtained from cryo-EM data.

A, The DeepEMhancer post-processed cryo-EM map (*top*), and the refined models of the DDD and DDD-Ube2e2 complexes from cryo-EM data collections of the DDD, DDD-COP1 and DDD-Ube2e2-COP1-c-Jun complex (*bottom*). The better-resolved structures are shown in panels **B** and **C**. **B**, The model of DDD complex displayed from different views. Cartoon representation of DET1 is shown on the right. The grey region highlights unresolved claw density in the reconstruction, observed in the absence of Ube2e2 binding. **C**, The model of DDD-Ube2e2 complex is shown in various views. Cartoon representation of DET1 is presented on the right.

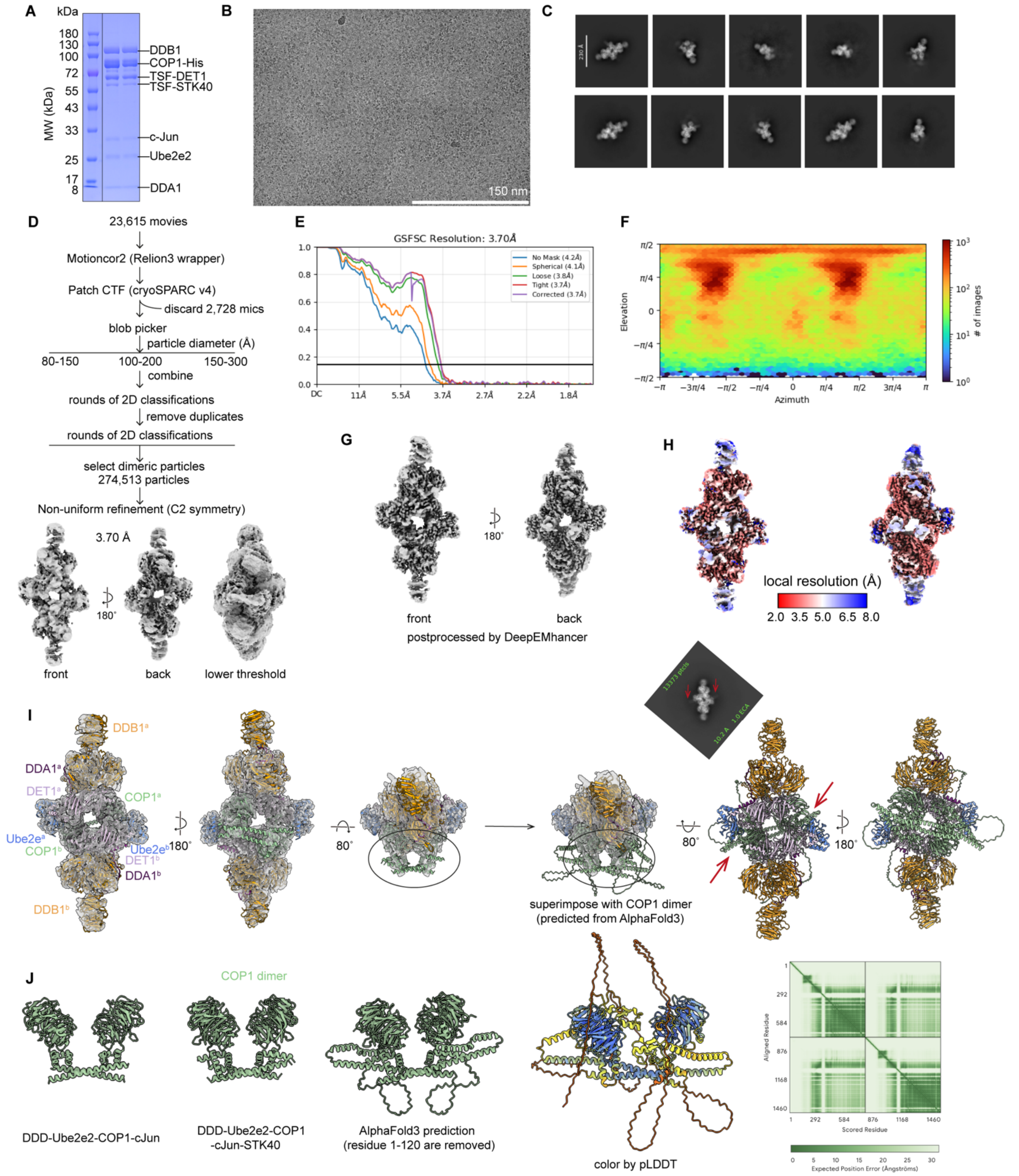

Supplementary Figure 11. Cryo-EM structure determination of the DDD-Ube2e2-COP1-c-Jun-STK40 complex.

A, Coomassie blue-stained SDS-PAGE analysis of the purified DDD-Ube2e2-COP1-c-Jun-STK40 complex. MW, molecular weight. TSF, twin-strep-FLAG. Source data are provided as a Source Data file. **B**, Representative cryo-EM micrograph of the DDD-Ube2e2-COP1-c-Jun-STK40 complex. Scale bar, 150 nm. **C**, Representative 2D class averages of the DDD-Ube2e2-COP1-c-Jun-STK40 complex.

D, Flow chart of cryo-EM data processing. **E**, FSC plots between two independently refined half-maps with no mask (blue), loose mask (green), tight mask (red), and corrected (purple). A cut-off of 0.143 (black line) was used to estimate the resolution. **F**, Angular particle distribution calculated in cryoSPARC for particle projections. The heatmap shows the number of particles for each viewing angle. **G**, Cryo-EM density map of DDD-Ube2e2-COP1-c-Jun-STK40 complex post-processed by DeepEMhancer. **H**, Local resolution map, colored-coded as indicated by the scale. **I**, The coordinate file of the DDD-Ube2e2-COP1-c-Jun-STK40 complex fitted into the cryo-EM map, presented from various views. The predicted COP1 dimer was further fitted into the experimental maps, and the resulting model shows the partial coiled-coil domain protruding in a manner similar to that observed in the 2D class averages. **J**, The COP1 dimer resolved from the DDD-Ube2e2-COP1-c-Jun and DDD-Ube2e2-COP1-c-Jun-STK40 experimental data is similar to the predicted model, which is further colored by predicted local distance difference test (pLDDT) as well as the predicted aligned error (PAE) plot shown on the right.

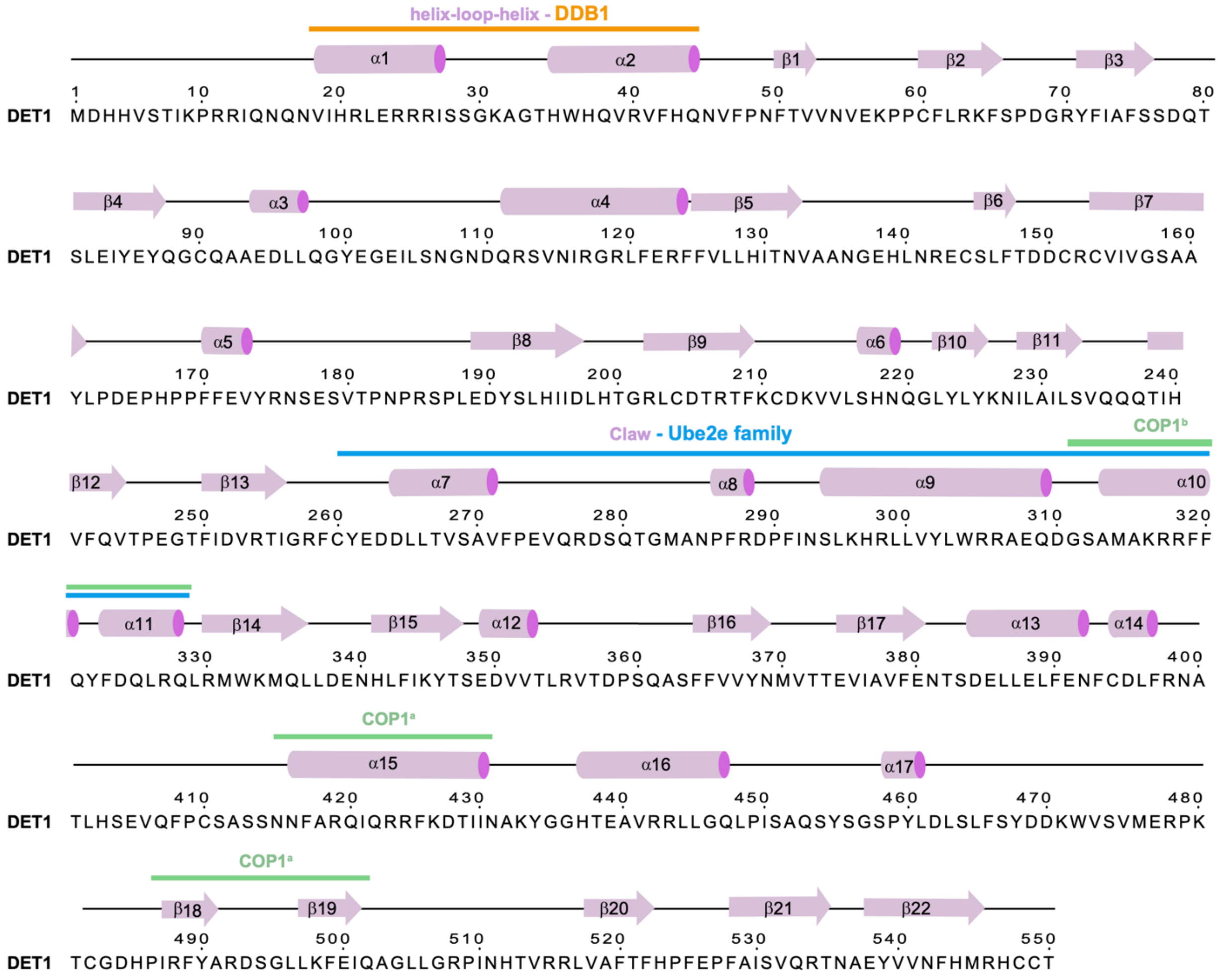

Supplementary Figure 12. DET1 sequence.

The human DET1 amino acid sequence is shown with residue numbering indicated above the sequence. Secondary structure elements based on the refined DDB1-DDA1-DET1-Ube2e2-COP1-c-Jun complex model are indicated above the sequence.

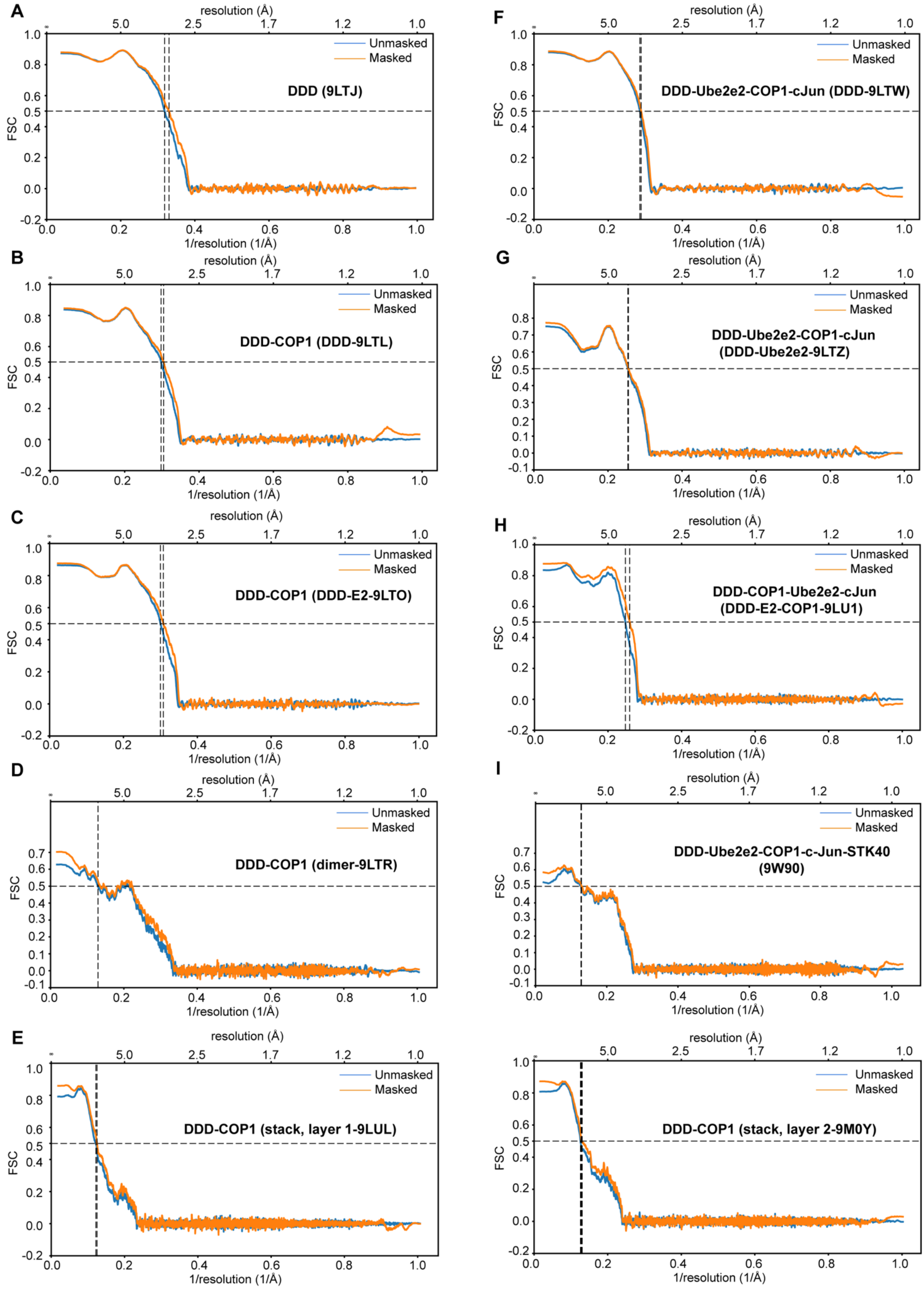
A
DDD (9LTJ)
B
DDD-COP1 (DDD-9LTL)
C
DDD-COP1 (DDD-E2-9LTO)
D
DDD-COP1 (dimer-9LTR)
E
DDD-COP1 (stack, layer 1-9LUL)
F
DDD-Ube2e2-COP1-cJun (DDD-9LTW)
G
DDD-Ube2e2-COP1-cJun
(DDD-Ube2e2-9LTZ)
H
DDD-COP1-Ube2e2-cJun
(DDD-E2-COP1-9LU1)
I
DDD-Ube2e2-COP1-c-Jun-STK40
(9W90)
DDD-COP1 (stack, layer 2-9M0Y)
Unmasked
Masked
resolution (Å)
FSC
1/resolution (1/Å)

Supplementary Figure 13. Map versus model FSC curves. Unsharpened maps were used for real-space refinements.

A, FSC curve comparing the model and cryo-EM map for the DDD complex. **B**, FSC curve comparing the model and cryo-EM map for the DDD-COP1 (DDD) complex. **C**, FSC curve comparing the model and cryo-EM map for the DDD-COP1 (DDD-E2) complex. **D**, FSC between the model of the DDD-COP1 (dimer) complex against the cryo-EM map. **E**, FSC between the layer 1 (*left*) and layer 2 (*right*) model of the DDD-COP1 (stack) against the cryo-EM maps. **F**, FSC between the model of the DDD-COP1-Ube2e2-c-Jun (DDD) complex against the cryo-EM map. **G**, FSC between the model of the DDD-COP1-Ube2e2-c-Jun (DDD-E2) against the cryo-EM map. **H**, FSC between the model and map for DDD-COP1-Ube2e2-c-Jun (DDD-Ube2e2-COP1) against the cryo-EM map. **I**, FSC between the model and map for DDD-COP1-Ube2e2-c-Jun-STK40 against the cryo-EM map.

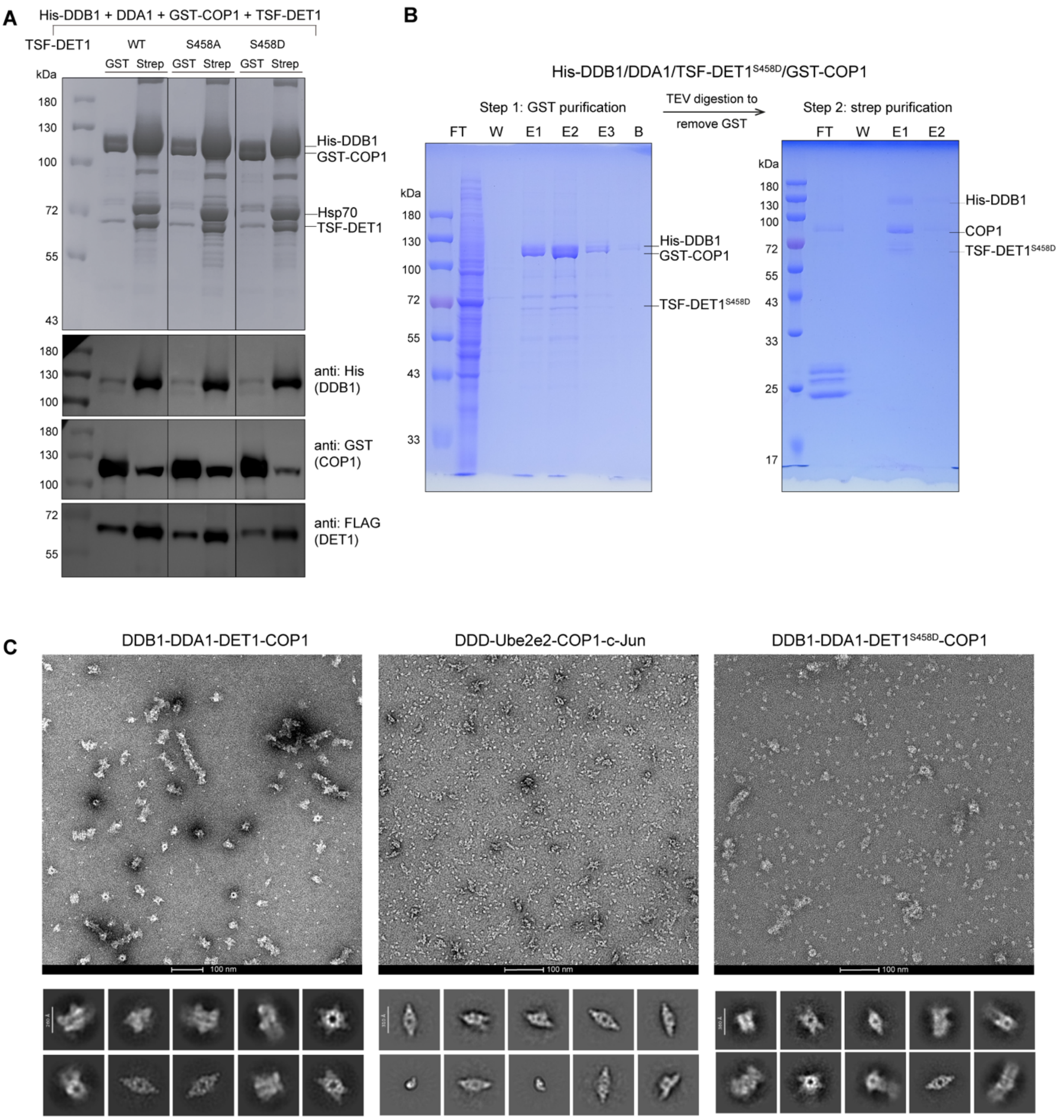

Supplementary Figure 14. Phosphomimetic S458D mutation of DET1 does not affect DET1 binding to COP1.

A, Pull-down experiment of wild-type or S458A/S458D TSF-DET1 mutant in cells co-transfected with GST-COP1, His-DDB1 and DDA1. Cell lysates were divided in two fractions and incubated with GST and strep beads. The eluents were analyzed by SDS-PAGE and western blotting. TSF, twin-strep-FLAG tag. Data in A are representative of three independent experiments. Source data are provided as a Source Data file. **B**, Tandem affinity purification of GST-COP1, His-DDB1, DDA1 and TSF-DET1^{S458D}. The target protein complex was purified by GST affinity chromatography (*left*), followed by overnight TEV digestion, and subsequent strep affinity purification (*right*). **C**, The negative stain micrographs of DDD-COP1 complex, DDD-Ube2e2-COP1-c-Jun and DDB1-DDA1- DET1^{S458D}-COP1 strep elution. Scale bar, 100 nm.

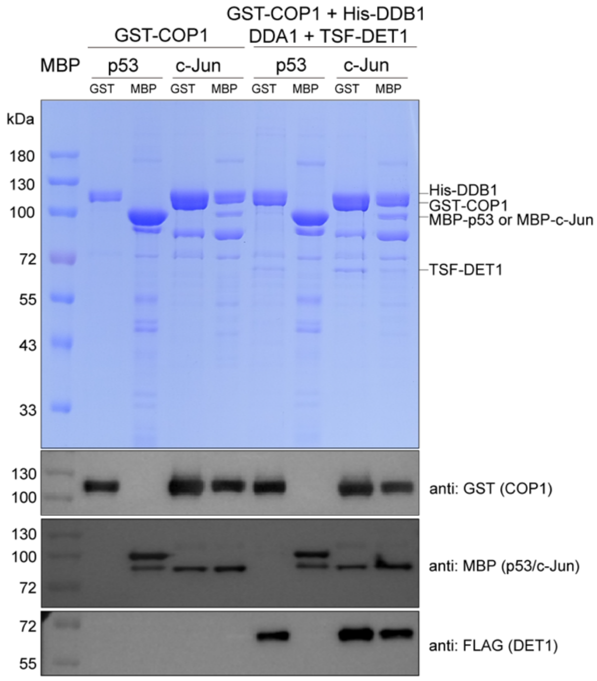

Supplementary Figure 15. Pull-down experiment of MBP-p53 or MBP-c-Jun in cells co-transfected with GST-COP1 or GST-COP1, His-DDB1, TSF-DET1 and DDA1.

Cell lysates were divided in two fractions and incubated with GST and MBP beads. The eluents were analyzed by SDS-PAGE and western blotting. TSF, twin-strep-FLAG tag. Data are representative of three independent experiments. Source data are provided as a Source Data file.

Supplementary Table 1. Cryo-EM data collection statistics for the DDD complex datasets.

	(EMD-63371)/(PDB 9LTJ)
	DDB1-DDA1-DET1
**Data collection and processing**	
Magnification	105,000 X
Voltage (kV)	300
Electron exposure ($e^{-}/Å^2$)	52.6
Defocus range (μm)	-1.2 to -1.8
Pixel size (Å)	0.85
Symmetry imposed	C1
Initial particle images (no.)	~4,800,000
Final particle images (no.)	~1,000,000
Map resolution (Å)<br>FSC threshold	2.65
**Refinement**	
Initial model used (PDB code)	6DSZ, DET1 (AF-Q7L5Y6-F1)
Model composition<br>Non-hydrogen atoms<br>Protein residues	 8,861<br>1,220
B factors ($Å^2$)<br>Protein (min/max/mean)	 78.65/414.95/150.61
R.m.s. deviations<br>Bond lengths (Å)<br>Bond angles (°)	 0.003<br>0.558
Validation<br>MolProbity score<br>Clashscore<br>Poor rotamers (%)	 1.75<br>7.50<br>0.12
Ramachandran plot<br>Favored (%)<br>Allowed (%)<br>Disallowed (%)	 95.05<br>4.87<br>0.08
CC (mask)<br>CC (box)<br>CC (peaks)<br>CC (volume)	0.86<br>0.86<br>0.77<br>0.85

Supplementary Table 2. Cryo-EM data collection statistics for the DDD-COP1 complex datasets.

	(EMD-63372)/ (PDB 9LTL)	(EMD-63374)/ (PDB 9LTO)	(EMD-63375)/ (PDB 9LTR)	(EMD-63397)/ (PDB 9LUL)	(EMD-63565)/ (PDB 9M0Y)
	DDB1-DDA1-DET1-COP1				
	DDB1-DDA1-DET1	DDB1-DDA1-DET1-E2	Dimer	Stack	
**Data collection and processing**					
Magnification	105,000 X				
Voltage (kV)	300				
Electron exposure (e⁻/Å²)	46.55, 46.32, 47.42, 48.60				
Defocus range (μm)	-1.0 to -1.8				
Pixel size (Å)	0.85				
Symmetry imposed	C1	C1	C2	C2 (layer 1)	C2 (layer 2)
Initial particle images (no.)	~1,780,000				
Final particle images (no.)	401,370	705,861	61,817	66,513	
Map resolution (Å)<br>FSC threshold	2.93	2.92	3.03	4.33	4.23
**Refinement**					
Initial model used (PDB code)	6DSZ, DET1 (AF-Q7L5Y6-F1)	6DSZ, 6W9A, DET1 (AF-Q7L5Y6-F1)	6DSZ, 6W9A, DET1 (AF-Q7L5Y6-F1), COP1 (AF-Q8NHY2-F1)	6DSZ, 6W9A, DET1 (AF-Q7L5Y6-F1), COP1 (AF-Q8NHY2-F1)	
Model composition<br>Non-hydrogen atoms<br>Protein residues	8,055<br>1,121	10,873<br>1,498	21,139<br>4,276	35,663<br>6,909	36,010<br>6,909
B factors (Å^2)<br>Protein (min/max/mean)	58.13/251.85/125.94	51.29/375.55/138.43	73.14/729.77/230.70	554.74/1319.39/829.98	530.07/1235.65/787.35
R.m.s. deviations<br>Bond lengths (Å)<br>Bond angles (°)	0.003<br>0.596	0.003<br>0.619	0.001<br>0.432	0.002<br>0.426	0.002<br>0.513
Validation<br>MolProbity score<br>Clashscore<br>Poor rotamers (%)	1.95<br>8.71<br>0.00	1.81<br>8.48<br>0.40	1.47<br>3.35<br>0.00	1.23<br>4.57<br>0.00	1.41<br>5.54<br>0.00
Ramachandran plot<br>Favored (%)<br>Allowed (%)<br>Disallowed (%)	92.00<br>7.82<br>0.18	94.92<br>5.08<br>0.00	95.01<br>4.87<br>0.12	98.05<br>1.94<br>0.01	97.45<br>2.46<br>0.09
CC (mask)<br>CC (box)<br>CC (peaks)<br>CC (volume)	0.79<br>0.85<br>0.69<br>0.78	0.81<br>0.86<br>0.72<br>0.81	0.54<br>0.73<br>0.30<br>0.59	0.56<br>0.77<br>0.25<br>0.50	0.60<br>0.82<br>0.34<br>0.51

Supplementary Table 3. Cryo-EM data collection statistics for DDD-COP1-Ube2e2$^{S76G-C139S}$-c-Jun complex datasets.

	(EMD-63383)/ (PDB 9LTW)	(EMD-63385)/ (PDB 9LTZ)	(EMD-63386)/ (PDB 9LU1)
	DDB1-DDA1-DET1-COP1-Ube2e2$^{S76G-C139S}$ -c-Jun		
	DDB1-DDA1-DET1	DDB1-DDA1-DET1-Ube2e2$^{S76G-C139S}$	DDB1-DDA1-DET1-Ube2e2$^{S76G-C139S}$-2COP1
**Data collection and processing**			
Magnification	105,000 X		
Voltage (kV)	300		
Electron exposure (e^-/Å^2)	48.89		
Defocus range (μm)	-1.2 to -1.8		
Pixel size (Å)	0.85		
Symmetry imposed	C1		
Initial particle images (no.)	~11,500,000	~11,500,000	~1,360,000
Final particle images (no.)	494,668	1,019,651	42,109
Map resolution (Å)<br>FSC threshold	3.25	3.26	3.62
**Refinement**			
Initial model used (PDB code)	6DSZ, DET1 (AF-Q7L5Y6-F1),	6DSZ, 6W9A, DET1 (AF-Q7L5Y6-F1),	6DSZ, 6W9A, DET1 (AF-Q7L5Y6-F1), COP1 (AF-Q8NHY2-F1)
Model composition<br>Non-hydrogen atoms<br>Protein residues	 8,004<br>1,138	 7,962<br>1,277	 17,116<br>2589
B factors (Å^2)<br>Protein (min/max/mean)	40.50/379.55/108.63	35.74/862.62/166.08	61.65/382.48/136.55
R.m.s. deviations<br>Bond lengths (Å)<br>Bond angles (°)	 0.003<br>0.567	 0.007<br>0.970	 0.003<br>0.520
Validation<br>MolProbity score<br>Clashscore<br>Poor rotamers (%)	 1.77<br>6.40<br>0.44	 2.03<br>9.29<br>0.21	 1.76<br>6.18<br>0.08
Ramachandran plot<br>Favored (%)<br>Allowed (%)<br>Disallowed (%)	 93.59<br>6.32<br>0.09	 90.53<br>9.39<br>0.08	 93.68<br>5.96<br>0.36
CC (mask)<br>CC (box)<br>CC (peaks)<br>CC (volume)	0.85<br>0.87<br>0.78<br>0.84	0.69<br>0.77<br>0.55<br>0.69	0.84<br>0.87<br>0.75<br>0.82

Supplementary Table 4. Cross-linked peptides identified in the DDD-Ube2e2-COP1-c-Jun complex.

Id	Score	Protein 1	PepPos1	PepSeq1	Link Pos1	Protein2	PepPos2	PepSeq2	Link Pos2
1	63.58	Ube2e2	34	EQVQPKKK	6	Ube2e2	42	EGKISSK	3
2	52.68	Ube2e2	194	QWTKR	4	DDA1	67	NAAKKR	4
3	78.17	COP1	506	SKVYQEHEKR	2	Ube2e2	45	ISSKTAAK	4
4	85.91	COP1	508	VYQEHEKR	7	c-Jun	264	IAASKCR	5
5	42.77	c-Jun	264	IAASKCR	5	c-Jun	253	IKAER	2
6	47.4	Ube2e2	34	EQVQPKKK	6	Ube2e2	49	TAAKLSTSAK	4
7	81.67	Ube2e2	45	ISSKTAAK	4	Ube2e2	53	LSTSAKR	6
8	40.33	Ube2e2	34	EQVQPKK	6	Ube2e2	53	LSTSAKR	6
9	63.05	COP1	506	SKVYQEHEKR	9	c-Jun	280	LEEKVK	4
10	43.63	Ube2e2	53	LSTSAKR	6	Ube2e2	42	EGKISSK	3
11	40.91	c-Jun	253	IKAER	2	c-Jun	284	VKTLK	2
12	122.76	Ube2e2	49	TAAKLSTSAKR	4	Ube2e2	42	EGKISSK	3
13	50.62	COP1	600	KAVSYAK	1	c-Jun	253	IKAER	2
14	100.21	DDA1	58	YLHQQWDKK	8	DDA1	67	NAAKKR	4
15	70.86	c-Jun	280	LEEKVK	4	c-Jun	253	IKAER	2
16	50.31	COP1	685	SVLDKDR	5	DDA1	66	KNAAK	1
17	64.08	c-Jun	264	IAASKCR	5	COP1	200	FEEKR	4
18	106.91	COP1	506	SKVYQEHEKR	2	COP1	635	SFKGHINEK	3
19	78.16	COP1	600	KAVSYAK	1	c-Jun	264	IAASKCR	5
20	62.3	COP1	635	SFKGHINEK	3	c-Jun	253	IKAER	2
21	45.11	COP1	685	SVLDKDR	5	DDA1	67	NAAKK	4
22	58.11	c-Jun	264	IAASKCR	5	c-Jun	284	VKTLK	2
23	101.25	c-Jun	264	IAASKCR	5	c-Jun	280	LEEKVK	4
24	151.17	COP1	506	SKVYQEHEKR	9	COP1	528	LLASGSDDAKVK	10
25	71.65	COP1	635	SFKGHINEK	3	c-Jun	264	IAASKCR	5
26	98.37	DDA1	71	KRDQEQVELEGESSAPPR	1	DDA1	66	KNAAK	1
27	57.71	c-Jun	280	LEEKVK	4	COP1	200	FEEKR	4
28	100.21	c-Jun	280	LEEKVK	4	c-Jun	284	VKTLK	2
29	93.6	COP1	635	SFKGHINEK	3	COP1	600	KAVSYAK	1
30	81.69	COP1	205	FKLDHSVSSTNGHR	2	COP1	198	QRFEEKR	6
31	77.37	DDB1	199	EKEFNKGPWK	6	DDA1	67	NAAKK	4
32	70.86	DET1	425	FKDTIINAK	2	DDA1	66	KNAAK	1
33	81.69	DET1	425	FKDTIINAK	2	DDA1	67	NAAKK	4
34	77.93	DDA1	58	YLHQQWDKK	8	DDB1	199	EKEFNK	2
35	45.11	DDA1	71	KRDQEQVELEGESSAPPR	1	Ube2e2	194	QWTKR	4
36	41.96	DDA1	58	YLHQQWDKK	8	Ube2e2	194	QWTKR	4
37	40.91	COP1	587	NTKQPIMVFK	3	c-Jun	253	IKAER	2
38	48.17	COP1	345	KQPWYNSTLASR	1	COP1	635	SFKGHINEK	3
39	71.38	COP1	587	NTKQPIMVFK	3	c-Jun	264	IAASKCR	5
40	112.31	c-Jun	286	TLKAQNSELASTANMLR	3	c-Jun	280	LEEKVK	4
41	96.05	COP1	587	NTKQPIMVFK	3	COP1	528	LLASGSDDAKVK	10

42	48.67	COP1	587	NTKQPIMVFK	3	c-Jun	280	LEEKVK	4
43	55.57	DDB1	1064	SVGKIEHSFW R	4	DDB1	148	DNKELK	3
44	118.06	COP1	670	GLSKTLLTFK	4	COP1	635	SFKGHINEK	3
45	59.03	COP1	624	LWNVGKPYCL R	6	c-Jun	264	IAASKCR	5
46	72.35	COP1	624	LWNVGKPYCL R	6	COP1	587	NTKQPIMVFK	3

Supplementary Table 5. Cryo-EM data collection statistics for DDD-Ube2e2-COP1-c-Jun-STK40 complex datasets.

	(EMD-65758)/(PDB 9W90)
	DDD-Ube2e2-COP1-c-Jun-STK40
**Data collection and processing**	
Magnification	105,000 X
Voltage (kV)	300
Electron exposure ($e^{-}/Å^2$)	50
Defocus range (μm)	-1.5 to -2.3
Pixel size (Å)	0.83
Symmetry imposed	C2
Initial particle images (no.)	11,147,804
Final particle images (no.)	274,513
Map resolution (Å)<br>FSC threshold	3.70
**Refinement**	
Initial model used (PDB code)	6DSZ, DET1 (AF-Q7L5Y6-F1), COP1 (AF-Q8NHY2-F1)
Model composition<br>Non-hydrogen atoms<br>Protein residues	 25,082<br>4,541
B factors ($Å^2$)<br>Protein (min/max/mean)	 39.02/1011.95/254.58
R.m.s. deviations<br>Bond lengths (Å)<br>Bond angles (°)	 0.002<br>0.608
Validation<br>MolProbity score<br>Clashscore<br>Poor rotamers (%)	 1.81<br>8.82<br>0.39
Ramachandran plot<br>Favored (%)<br>Allowed (%)<br>Disallowed (%)	 95.14<br>4.79<br>0.07
CC (mask)<br>CC (box)<br>CC (peaks)<br>CC (volume)	0.48<br>0.68<br>0.21<br>0.61

Supplementary Table 6. Orientation Diagnostics of the structures reported in this study.

Structure	conical FSC area ratio (cFAR)	sampling compensation factor (SCF)
DDB1-DDA1-DET1 (EMD-63371)/ (PDB 9LTJ)	0.26	0.274
DDB1-DDA1-DET1 (EMD-63372)/(PDB 9LTL)	0.05	0.555
DDB1-DDA1-DET1-E2 (EMD-63374)/(PDB 9LTO)	0.10	0.534
DDB1-DDA1-DET1-COP1 dimer (EMD-63375)/(PDB 9LTR)	0.01	0.834
Stack (EMD-63397)/(PDB 9LUL); (EMD-63565)/(PDB 9M0Y)	0.08/0.07	0.809/0.765
DDB1-DDA1-DET1 (EMD-63383)/(PDB 9LTW)	0.20	0.591
DDB1-DDA1-DET1-Ube2e2$^{S76G-C139S}$ (EMD-63385)/(PDB 9LTZ)	0.08	0.579
DDB1-DDA1-DET1-Ube2e2$^{S76G-C139S}$-2COP1 (EMD-63386)/(PDB 9LU1)	0.14	0.728
DDD-Ube2e2-COP1-c-Jun-STK40 (EMD-65758)/ (PDB 9W90)	0.02	0.847
