# Supplementary material for: Cryo-EM structure of the human COP1-DET1 ubiquitin ligase complex
Source: Nat Commun. 2026 Jan 15;17:543. doi: 10.1038/s41467-026-68375-7 (PMC12808801; doi:10.1038/s41467-026-68375-7)
Supplement: Supplementary file 3 — Reporting Summary [file 41467_2026_68375_MOESM3_ESM.pdf]

nature portfolio

Corresponding author(s): Ming-Yuan Su

Last updated by author(s): Dec 08, 2025

Reporting Summary

Nature Portfolio wishes to improve the reproducibility of the work that we publish. This form provides structure for consistency and transparency in reporting. For further information on Nature Portfolio policies, see our Editorial Policies and the Editorial Policy Checklist.

Statistics

For all statistical analyses, confirm that the following items are present in the figure legend, table legend, main text, or Methods section.

n/a	Confirmed	
☐	☒	The exact sample size (*n*) for each experimental group/condition, given as a discrete number and unit of measurement
☐	☒	A statement on whether measurements were taken from distinct samples or whether the same sample was measured repeatedly
☒	☐	The statistical test(s) used AND whether they are one- or two-sided<br>*Only common tests should be described solely by name; describe more complex techniques in the Methods section.*
☒	☐	A description of all covariates tested
☒	☐	A description of any assumptions or corrections, such as tests of normality and adjustment for multiple comparisons
☒	☐	A full description of the statistical parameters including central tendency (e.g. means) or other basic estimates (e.g. regression coefficient) AND variation (e.g. standard deviation) or associated estimates of uncertainty (e.g. confidence intervals)
☒	☐	For null hypothesis testing, the test statistic (e.g. *F*, *t*, *r*) with confidence intervals, effect sizes, degrees of freedom and *P* value noted<br>*Give P values as exact values whenever suitable.*
☒	☐	For Bayesian analysis, information on the choice of priors and Markov chain Monte Carlo settings
☒	☐	For hierarchical and complex designs, identification of the appropriate level for tests and full reporting of outcomes
☒	☐	Estimates of effect sizes (e.g. Cohen's *d*, Pearson's *r*), indicating how they were calculated

Our web collection on statistics for biologists contains articles on many of the points above.

Software and code

Policy information about availability of computer code

Data collection	SerialEM (3.8.0) and EPU are used for cryo-EM data collection. Crosslinking mass spectrometry (XL-MS) uses Easy-nLC 1000 system and Orbitrap Fusion mass spectrometer (Thermo Fisher). For protein identification, peptides were ionized via electrospray ionization and analyzed by Orbitrap Eclipse (Thermo Fisher) mass spectrometer using data dependent tandem MS/MS acquisition.
Data analysis	For Cryo-EM data processing, the following software were used: MotionCor2, Relion 3, cryoSPARC v4;<br>For model building and analysis, the following software were used: Coot 0.8.9.3, Phenix 1.20.1-4487, UCSF Chimera 1.15, ChimeraX 1.4, DeepEMhancer 'tightTarget' model, AlphaFold2 and AlphaFold3;<br>Graphs plotting: OriginPro 2021pro;<br>Cross-linked peptides were analyzed and identified using Proteome Discoverer 2.4 and XlinkX software 2.4 (Thermo Fisher);<br>For protein identification, the MS/MS spectra were searched against the human uniport database using the Proteome Discoverer 2.5 software.

For manuscripts utilizing custom algorithms or software that are central to the research but not yet described in published literature, software must be made available to editors and reviewers. We strongly encourage code deposition in a community repository (e.g. GitHub). See the Nature Portfolio guidelines for submitting code & software for further information.

Data

Policy information about availability of data

All manuscripts must include a data availability statement. This statement should provide the following information, where applicable:

- Accession codes, unique identifiers, or web links for publicly available datasets
- A description of any restrictions on data availability
- For clinical datasets or third party data, please ensure that the statement adheres to our policy

The cryo-EM density maps have been deposited in the Electron Microscopy Data Bank (EMDB) under accession codes EMD-63371, EMD-63372, EMD-63374, EMD-63375, EMD-63397, EMD-63383, EMD-63385, EMD-63386, EMD-63365 and EMD-65758. The corresponding coordinates were deposited in the Protein Data Bank under accession numbers 9LTJ, 9LTL, 9LTO, 9LTR, 9LUL, 9LTW, 9LTZ, 9LU1, 9M0Y and 9W90. Previously solved structures used in this study were obtained from the PDB with accession codes: 6FGA [https://www.rcsb.org/structure/6FGA], 6S53 [https://www.rcsb.org/structure/6S53], 5IGQ [https://www.rcsb.org/structure/5IGQ], 8WQR [https://www.rcsb.org/structure/8WQR], 8TL6 [https://www.rcsb.org/structure/8TL6], 8AJN [https://www.rcsb.org/structure/8AJN], 3BZH [https://www.rcsb.org/structure/3BZH], 1X23 [https://www.rcsb.org/structure/1X23], 6DSZ [https://www.rcsb.org/structure/6DSZ], 6W9A [https://www.rcsb.org/structure/6W9A]. The mass spectrometry proteomics data were deposited to the ProteomeXchange Consortium through the PRIDE partner repository with the dataset identifier PXD071633.

Research involving human participants, their data, or biological material

Policy information about studies with human participants or human data. See also policy information about sex, gender (identity/presentation), and sexual orientation and race, ethnicity and racism.

Reporting on sex and gender	N/A
Reporting on race, ethnicity, or other socially relevant groupings	N/A
Population characteristics	N/A
Recruitment	N/A
Ethics oversight	N/A

Note that full information on the approval of the study protocol must also be provided in the manuscript.

Field-specific reporting

Please select the one below that is the best fit for your research. If you are not sure, read the appropriate sections before making your selection.

☒ Life sciences ☐ Behavioural & social sciences ☐ Ecological, evolutionary & environmental sciences

For a reference copy of the document with all sections, see nature.com/documents/nr-reporting-summary-flat.pdf

Life sciences study design

All studies must disclose on these points even when the disclosure is negative.

Sample size	No statistical methods were used to predetermine sample size. The sample size was determined based on sufficient EM data that can achieve adequate single-particle EM analysis and 3D cryo-EM reconstructions.
Data exclusions	Electron microscopy: micrographs were screened and bad ones discarded. After CTF estimation, the bad movies which were bad focus or included ice contamination were excluded. For protein identification in mass spectrometry experiments, several hits were excluded since they were carryover from previous LC-MS runs.
Replication	For each experiment, at least three replicates are done. All replicates were successful and yielded similar results.
Randomization	Randomization is not relevant to this study since no animals or human research participants are involved in this research. There is not requirement to randomize the data.
Blinding	Blinding is not relevant to this study since no human or animals are involved in this study. We follow generally accepted protocol.

Reporting for specific materials, systems and methods

We require information from authors about some types of materials, experimental systems and methods used in many studies. Here, indicate whether each material, system or method listed is relevant to your study. If you are not sure if a list item applies to your research, read the appropriate section before selecting a response.

Materials & experimental systems

n/a	Involved in the study
☐	☒ Antibodies
☐	☒ Eukaryotic cell lines
☒	☐ Palaeontology and archaeology
☒	☐ Animals and other organisms
☒	☐ Clinical data
☒	☐ Dual use research of concern
☒	☐ Plants

Methods

n/a	Involved in the study
☒	☐ ChIP-seq
☒	☐ Flow cytometry
☒	☐ MRI-based neuroimaging

Antibodies

Antibodies used

Anti-MBP tag Mouse Monoclonal antibody and anti c-Jun Rabbit Monoclonal antibody was used at 1:1000 dilution (Beyotime, AF2912, AF1612), anti ubiquitin was used at 1:20000 dilution (Abcam, ab134953), anti-GST tag Mouse Monoclonal antibody was used at 1:5000 dilution (Yeason, 30901ES50), HRP-conjuagated FLAG-tag Mouse Monoclonal antibody was used at 1:1000 dilution (Yeason, 30502ES60). The secondary antibodies HRP Conjugated Goat anti-Mouse IgG was used at 1:5000 dilution (CWBIO, CW0102S), HRP Conjugated Goat anti-Rabbit IgG was used at 1:5000 dilution (CWBIO, CW0103S).

Validation

All antibodies used in this study are commercially available and widely used in publications.
Anti-MBP tag Mouse Monoclonal antibody (https://www.beyotime.com/product/AF2912.htm);
Anti c-Jun Rabbit Monoclonal antibody (https://www.beyotime.com/product/AF1612.htm);
Anti ubiquitin antibody (https://www.abcam.cn/products/primary-antibodies/ubiquitin-antibody-epr8830-ab134953.html);
Anti-GST tag Mouse Monoclonal antibody (https://www.yeasen.com/products/detail/165);
HRP-conjuagated FLAG-tag Mouse Monoclonal antibody (https://www.yeasen.com/products/detail/88);
HRP Conjugated Goat anti-Mouse IgG (https://www.cwbio.com/product/detail/id/10118);
HRP Conjugated Goat anti-Rabbit IgG (https://www.cwbio.com/product/detail/id/10119).

Eukaryotic cell lines

Policy information about cell lines and Sex and Gender in Research

Cell line source(s)

Expi293F cell (Thermo Fisher, Cat#A14527CN)

Authentication

Expi293F cell lines used were not authenticated.

Mycoplasma contamination

Expi293F cell lines were not tested for mycoplasma contamination.

Commonly misidentified lines
(See ICLAC register)

No commonly misidentified cell lines were used.

Plants

Seed stocks

N/A

Novel plant genotypes

N/A

Authentication

N/A
